# Role of Phospholipase A_2_ in Retrograde Transport of Ricin

**DOI:** 10.3390/toxins3091203

**Published:** 2011-09-23

**Authors:** Tove Irene Klokk, Anne Berit Dyve Lingelem, Anne-Grethe Myrann, Kirsten Sandvig

**Affiliations:** 1 Centre for Cancer Biomedicine, Department of Biochemistry, Institute for Cancer Research, The Norwegian Radium Hospital, Oslo University Hospital, Montebello, 0310 Oslo, Norway; Email: tove.irene.klokk@rr-research.no (T.I.K.); anne.berit.dyve@rr-research.no (A.B.D.L.); anne.grethe.myrann@rr-research.no (A.-G.M.); 2 Department of Molecular Biosciences, University of Oslo, 0316 Oslo, Norway

**Keywords:** ricin, retrograde transport, phospholipase A_2_, Golgi, toxin

## Abstract

Ricin is a protein toxin classified as a bioterror agent, for which there are no known treatment options available after intoxication. It is composed of an enzymatically active A-chain connected by a disulfide bond to a cell binding B-chain. After internalization by endocytosis, ricin is transported retrogradely to the Golgi and ER, from where the ricin A-chain is translocated to the cytosol where it inhibits protein synthesis and thus induces cell death. We have identified cytoplasmic phospholipase A_2_ (PLA_2_) as an important factor in ricin retrograde transport. Inhibition of PLA_2_ protects against ricin challenge, however the toxin can still be endocytosed and transported to the Golgi. Interestingly, ricin transport from the Golgi to the ER is strongly impaired in response to PLA_2_ inhibition. Confocal microscopy analysis shows that ricin is still colocalized with the *trans*-Golgi marker TGN46 in the presence of PLA_2_ inhibitor, but less is colocalized with the *cis*-Golgi marker GM130. We propose that PLA_2_ inhibition results in impaired ricin transport through the Golgi stack, thus preventing it from reaching the ER. Consequently, ricin cannot be translocated to the cytosol to exert its toxic action.

## 1. Introduction

Ricin is a very potent toxin isolated from the seeds of the castor oil plant *Ricinus communis*, and it is classified as a potential bioterror agent, for which no treatment is available [1]. Ricin is a protein toxin, consisting of two polypeptide chains A and B, linked via a disulfide bridge. The B-chain mediates the binding to glycolipids or glycoproteins with a terminal galactose at the cell surface, followed by endocytic uptake into the cell [2,3]. After endocytosis, a fraction of the internalized ricin is transported retrogradely from endosomes to the Golgi, and further on to the endoplasmic reticulum (ER). In the ER, the disulfide bridge between the A- and B-chain is reduced, and the enzymatically active A-chain is translocated to the cytosol where it inactivates the 28S ribosomal RNA and subsequently inhibits protein synthesis and induces cell death (for review see e.g., [4]). As there is no proven, safe treatment for ricin intoxication, the investigation into the molecular details of the uptake and transport of ricin into cells is highly important. It has been shown that inhibition of retrograde transport of ricin not only protects cells [5] but also mice [6] from ricin challenge, suggesting the retrograde route as a potential therapeutic target. Importantly, studies of the retrograde transport of ricin will contribute to our knowledge about this pathway.

The endocytosis and retrograde transport of toxins are complicated processes, regulated by a variety of factors (for reviews, see e.g., [4,7]). During the last decades, it has become evident that also lipids play a major role in toxin transport. For instance cholesterol has been shown to be important for the intracellular transport of both ricin and the bacterial toxin Shiga toxin (Stx) [8,9,10]. Furthermore, depletion of sphingolipids facilitates endosome to Golgi transport of ricin [11], and it has been demonstrated that the composition of glycosphingolipids in the cellular membrane is crucial for the uptake of Stx [12,13,14,15,16]. Polyunsaturated fatty acids also regulate Stx transport [17], and the general membrane bilayer composition of lipids play an important role in intracellular transport, as does membrane tubule formation [18,19]. It is therefore apparent that not only other proteins, but also lipids play an important role in uptake mechanisms and retrograde transport of protein toxins.

Phospholipase A_2_s (PLA_2_s) are enzymes catalyzing the hydrolysis of glycerophospholipids into lysophospholipids and free fatty acids [20]. The presence of lysophospholipids may increase the curvature on the cytosolic leaflet of organelle membranes, hence PLA_2_s are involved in both membrane shape formation and function [21,22]. In addition, membrane tubule formation seems to be dependent on PLA_2_ activity, as for instance the Brefeldin A (BFA)-stimulated induction of Golgi membrane tubulation [23,24]. It was recently demonstrated that PLA_2_ enzymes are involved in the earliest steps of membrane tubule formation at the *trans*-Golgi Network (TGN) [25]. Endosomal membrane tubulation has also been demonstrated to be dependent on PLA_2_ activity [26,27]. Furthermore, PLA_2_ is important for intracellular trafficking events such as recycling of transferrin (Tf) and low-density lipoprotein receptor (LDLR) [26,28], and the degradative pathway of LDL and epidermal growth factor (EGF) [28]. In addition, it has been demonstrated that PLA_2_ antagonists can block constitutive cycling of chimeric temperature-sensitive proteins between the Golgi and ER [29]. Most of these studies have been performed using small molecule inhibitors of PLA_2_ activity, such as ONO-RS-082 (ONO) and bromoenol lactone (BEL). However, the involvement of PLA_2_ activity in these processes has been further demonstrated by the stimulation of Golgi membrane tubulation and retrograde trafficking to the ER by PLA_2_ activating peptide (PLAP) [30] and by inhibition of LPAT (lysophosphatidylcholine acyltransferase), which catalyzes the reverse reaction of PLA_2_[31]. Furthermore, PLA_2_ activity is induced by the addition of cholesterol, and is involved in the cholesterol-induced vesiculation of the Golgi apparatus [32]. 

Given the importance of PLA_2_ activity in membrane tubulation and intracellular trafficking events, we decided to investigate the role of PLA_2_ in the retrograde transport of ricin. To this end we used the PLA_2_ antagonists ONO and BEL, and specially designed ricin molecules to study the intracellular trafficking of the toxin. The data clearly demonstrate a role of PLA_2_ activity in the retrograde transport of ricin. Treatment with the PLA_2_ inhibitor ONO protected the cells against ricin challenge, and ricin transport to the ER was strongly impaired. Ricin could still be endocytosed and transported to the TGN, suggesting that it was the TGN-to-ER transport step that was dependent on PLA_2_ activity. Altogether the data indicate that PLA_2_ is an important player in the retrograde transport of ricin.

## 2. Materials and Methods

### 2.1. Materials

All chemicals were purchased from Sigma-Aldrich (St. Louis, MO, USA) if not otherwise noted. H_2_^35^SO_4_ and [^3^H]Leucine were purchased from Hartman Analytic (Braunschweig, Germany), D-[2-^3^H(N)]-mannose and Na^125^I were from Perkin Elmer (Boston, MA, USA). ONO-RS-082 (ONO) was purchased from ENZO Life Sciences (Plymouth, PA, USA), and bromoenol lactone (BEL) from Sigma-Aldrich. Rabbit anti-ricin antibody was from Sigma Aldrich, sheep anti-TGN46 antibody was from AbD Serotec (Oxford, UK), and mouse anti-GM130 was from BD Bioscience (San Jose, CA, USA). Ricin-sulf-1 (RS1), ricin with a modified ricin A-subunit containing a tyrosine sulfation site, and ricin-sulf-2 (RS2), ricin with a modified ricin A-subunit containing a tyrosine sulfation site and three partially overlapping *N*-glycosylation sites in the *C*-terminal, was produced and purified as described earlier [33]. Plasmids encoding TPST-1-EGFP and TPST-2-EGFP were a kind gift from Dr. Ludger Johannes (Institut Curie, Paris, France).

### 2.2. Cell Culture and Transfection

HEp-2 cells were maintained in Dulbecco’s modified Eagle’s medium (Invitrogen, Carlsbad, CA, USA) supplemented with 10% fetal calf serum, 100 units/mL penicillin and 100 µg/mL streptomycin, at 37 °C under 5% CO_2_. For cytotoxicity and endocytosis assays, the cells were seeded out in 24-well plates at a density of 5 × 10^4^ cells/well one day before the experiment. For the sulfation and mannosylation assays, cells were seeded in 6-well plates at a density of 2 × 10^5^ cells/well, and for confocal microscopy studies the cells were seeded in 6-well plates with two coverslips per well at a density of 1 × 10^5^ cells/well, one day before the experiment. For transfection with pTPST-1-EGFP and pTPST-2-EGFP, cells on coverslips were transfected using Fugene 6 according to the manufacturer’s instructions (Roche Diagnostics, Mannheim, Germany) one day before use in confocal microscopy studies.

### 2.3. Cytotoxicity Assay

Cells grown in 24-well plates were washed twice in leucine-free HEPES-buffered medium before addition of inhibitors at indicated concentrations for 30 min. Increasing concentrations of ricin (1–1000 ng/mL) were added, and the cells incubated for 3 h at 37 °C. The medium was then replaced with leucine-free HEPES-buffered medium containing 2 µCi/mL [^3^H]Leucine, and the cells incubated further for 20 min. The proteins were precipitated with 5% (w/v) trichloracetic acid (TCA), washed once in 5% (w/v) TCA, and then dissolved in 0.1 M KOH. The incorporation of radioactively labeled leucine was quantified, and IC50 calculated as the concentration of toxin giving a 50% reduction in protein synthesis. Fold protection was then calculated as the ratio between IC50 for inhibitor-treated cells compared to control cells.

### 2.4. Sulfation Assay

The cells were washed twice with sulfate-free medium supplemented with 2 mM L-glutamine, followed by incubation with 0.2 mCi/mL H_2_^35^SO_4_ in sulfate-free medium for 2.5 h at 37 °C. The cells were then incubated with inhibitors at the indicated concentrations for 30 min before addition of ricin-sulf-1 (6 µg/mL) for an additional 2 h. The medium was removed and the cells washed 2 × 5 min with 0.1 M lactose in HEPES-buffered medium, and once in cold PBS on ice before addition of 400 µL lysis buffer [0.1 M NaCl, 10 mM Na2HPO4, 1 mM EDTA, 1% Triton X-100, 60 mM octyl glycopyranoside] supplemented with Complete protease inhibitors (Roche Diagnostics, Mannheim, Germany). The lysate was cleared by centrifugation (8000 rpm, 10 min) and ricin-sulf-1 was immunoprecipitated with anti-ricin antibody, pre-bound to protein A-sepharose beads (Amersham Biosciences, Buckinghamshire, UK), overnight at 4 °C. The precipitate was separated by SDS-PAGE under reducing conditions, and blotted onto a PVDF membrane (Immobilon-P, Millipore, Billerica, MA, USA). The bands were detected by autoradiography and imaged and quantified using PharosFX scanner and Quantity One^®^ 1-D Analysis Software (BioRad Laboratories Inc, Hercules, CA, USA). The total amount of sulfated proteins was determined by TCA-precipitation of all ^35^S-labeled proteins in the lysates.

### 2.5. Mannosylation Assay

The cells were washed twice with glucose-free DMEM, followed by incubation in glucose-free DMEM containing 1.8 µL/mL D-(+)-glucose solution (10%) and 0.1 mCi/mL D-[2-^3^H(N)]-mannose for 2.5 h at 37 °C. ONO was then added at indicated concentrations, in addition to 1 µg/mL swainsonine, and the cells incubated for another 30 min. Ricin-sulf-2 (6 µg/mL) was then added and incubation continued for another 2 h. Subsequently, the cells were treated following the sulfation assay protocol described above. The bands were detected using Kodak BioMax MS-film (Sigma-Aldrich), scanned and quantified by Quantity One^®^ 1-D Analysis Software (BioRad Laboratories Inc, Hercules, CA, USA). The total amount of mannosylated proteins was determined by TCA-precipitation of all ^3^H-labeled proteins in the lysates.

### 2.6. Endocytosis of ^125^I-ricin

Cells were pre-incubated with 2.5 or 5 µM ONO for 30 min, before addition of ^125^I-ricin for 2 h at 37 °C. Binding of ricin was measured as the amount of cell-associated ricin after washing with ice-cold buffer (0.14 M NaCl, 2 mM CaCl_2_, 20 mM HEPES; pH 7.0). Endocytosed ricin was measured as the amount of ^125^I-ricin that was not removed after 2 × 5 min washing with 0.1 M lactose in HEPES-buffered medium.

### 2.7. Confocal Microscopy

The cells grown on glass coverslips were washed once in HEPES-buffered medium before addition of 5 µM ONO for 30 min. Ricin-sulf-1 (~1 µg/mL) was added for 15 min, followed by 30 min chase. The cells were then washed twice with 0.1 M lactose in HEPES-buffered medium, and twice in PBS. The cells were fixed with 10% formalin-solution, and permeabilized in 0.1% Triton X-100. Blocking solution, 5% fetal calf serum in PBS, was then added for 30 min, before incubation with the appropriate primary and secondary antibodies. Fluorophore-labeled secondary antibodies were obtained from Jackson ImmunoResearch Laboratories (West Grove, PA, USA). The cells were mounted in Prolong^®^Gold containing DAPI for staining of the nuclei (Molecular Probes, Eugene, OR, USA), and imaged on laser scanning confocal microscope LSM 510 DUO (Carl Zeiss, Jena, Germany). Images were prepared and analyzed with the LSM Image Browser software (Carl Zeiss) or ImageJ software (NIH, Bethesda, USA). Colocalization was quantified using the LSM Image Examiner (Carl Zeiss), and presented as percentage colocalization compared to control.

## 3. Results

### 3.1. PLA_2_ Inhibitor Protects Cells against Ricin

**Figure 1:**
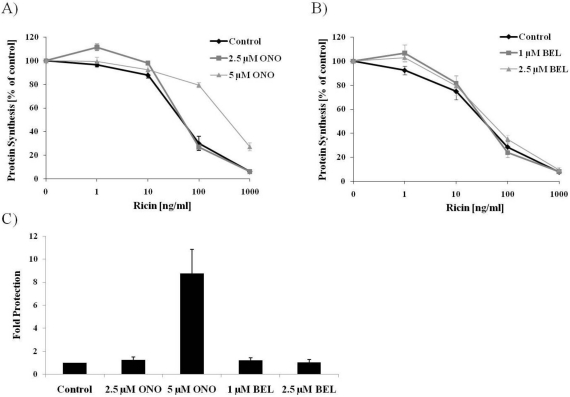
PLA_2_ inhibitor ONO protects against ricin challenge. HEp-2 cells were pre-treated with inhibitors ONO (**A**) or BEL (**B**) for 30 min before addition of ricin for 3 h followed by [^3^H]leucine for 20 min. Protein biosynthesis was measured by [^3^H]leucine incorporation, and toxicity curves were generated; (**C**) The fold protection against ricin was calculated at 50% inhibition of protein biosynthesis and compared to control which was set to 1. The average of at least three experiments is shown, and error bars indicate standard error of the mean (SEM).

In order to examine the potential involvement of PLA_2_ enzymes in the intracellular transport of ricin, we took advantage of the commonly used PLA_2_ inhibitors ONO-RS-082 (ONO) and bromoenol lactone (BEL). First we investigated if treatment with ONO had any effect on the overall cytotoxicity of ricin in HEp-2. The cells were pretreated with two different concentrations of ONO, before exposure to increasing concentrations of ricin. Toxin response curves were created, and as can be seen in Figure 1A, pre-treatment with 5 µM ONO had a significant protective effect against ricin. As a quantitative measure of this effect, the fold protection was calculated as the concentration of ricin giving a 50% reduction in protein synthesis in treated cells compared to untreated cells (Figure 1C), and 5 µM ONO resulted in an almost 9-fold protection against ricin. Cytoplasmic PLA_2_s can be divided into Ca^2+^-dependent and Ca^2+^-independent (iPLA_2_) enzymes [20], and ONO is an inhibitor of both of these classes. BEL is an irreversible inhibitor of iPLA_2_, and was also tested in ricin toxicity assays. At a concentration of 2.5 µM, which is commonly used to inhibit iPLA_2_ activity [26,29], BEL did not have any protective effect against ricin (Figure 1B,C). These data then suggest that it is the Ca^2+^-dependent PLA_2_ enzymes that are involved in the ricin intoxication process.

Shiga toxin (Stx) is a bacterial toxin which is also endocytosed and transported retrogradely from the Golgi to the ER, and finally the cytosol (see e.g., [34]). Interestingly, treatment with 2.5 or 5 µM ONO had no significant effect on the toxicity of Stx in these cells (Supplementary Figure S1A), indicating that Stx transport to the cytosol of HEp-2 cells is not dependent on PLA_2_ activity.

### 3.2. Ricin Sulfation Is Strongly Increased in Response to PLA_2_ Inhibition

**Figure 2:**
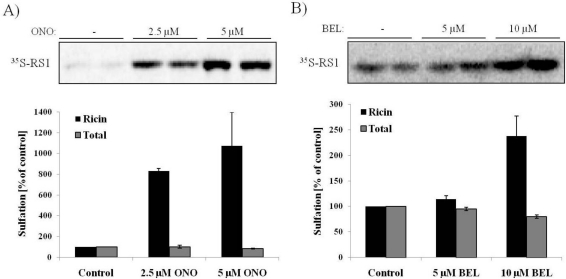
Increased sulfation in response to PLA_2_ inhibitors. HEp-2 cells were incubated with radioactive sulfate for 2.5 h before addition of ONO (**A**) or BEL (**B**) for 30 min and then ricin-sulf-1 for another 2 h. Ricin was immunoprecipitated from the lysates, and the precipitate separated by SDS-PAGE. The amount of sulfated ricin (^35^S-RS1) was visualized by autoradiography, and the relative change compared to control was quantified. The total amount of sulfated proteins was determined by TCA-precipitation of all ^35^S-labeled proteins in the lysates. The autoradiogram from one representative experiment is shown, and the bar graph shows the average of at least three experiments with error bars representing standard error of the mean (SEM).

Given the observed protection against ricin by the PLA_2_ inhibitor ONO, we were interested in determining which step in the retrograde transport pathway that was impaired in the presence of this compound. As mentioned in the introduction, PLA_2_ activity is important for endosomal membrane tubulation, and PLA_2_ enzymes are involved in several endosomal trafficking events [26,28]. It could therefore be speculated that ricin transport from endosomes to the Golgi could be impaired in response to PLA_2_ inhibition. In order to address this, we took advantage of a modified ricin molecule, ricin-sulf-1, with a tyrosine sulfation site attached *C*-terminally to the ricin A-chain. As sulfation is a modification that occurs mainly in the TGN [35], it is possible to measure the amount of internalized ricin reaching the Golgi apparatus using radioactively labeled sulfate. Interestingly, ricin transport to the Golgi is not impaired in the presence of inhibitors ONO (Figure 2A) or BEL (Figure 2B). Surprisingly, the amount of sulfated ricin was strongly increased in response to inhibitor treatment. Control experiments show that there was no effect on total protein sulfation in the cells. Quantification of the data shows that there is as much as an 8-fold increase in the amount of sulfated ricin-sulf-1 in response to 2.5 µM ONO and a 10-fold increase for 5 µM ONO. For BEL, the effect on ricin sulfation was weaker, but still giving a 2.5-fold increase in response to 10 µM BEL. These data indicate that ricin is being transported to the Golgi apparatus in presence of inhibitors, and they show that the degree of sulfation is actually increased after inhibitor treatment. The sulfation of Stx was not altered in response to ONO (Supplementary Figure S1B).

**Figure 3:**
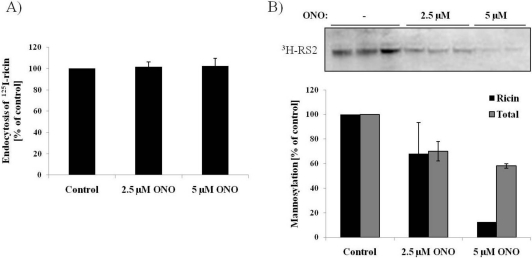
Reduced transport to the ER in presence of PLA_2_ inhibitor ONO. (**A**) After pre-treatment with the indicated concentrations of ONO for 30 min, ^125^I-ricin was added to the HEp-2 cells and incubation continued for 2 h. Endocytosed ricin was measured as the amount of ^125^I-ricin that was not removed after washing with 0.1 M lactose in HEPES-buffered medium. The average of three experiments is shown, with error bars representing standard error of the mean (SEM); (**B**) HEp-2 cells were incubated with radioactive mannose for 2.5 h before addition of ONO for 30 min and then ricin-sulf-2 for another 2 h. Ricin was immunoprecipitated from the lysates, and the precipitate separated by SDS-PAGE. The bands were detected using Kodak BioMax MS-film (Sigma-Aldrich), quantified and presented as percentage of mannosylated ricin compared to control. The experiment was repeated twice, and a representative experiment is shown. The total amount of mannosylated proteins was determined by TCA-precipitation of all ^3^H-labeled proteins in the lysates.

Increased ricin sulfation could be explained by an increase in the internalization of the toxin. In order to examine this possibility, endocytosis of ^125^I-labelled ricin was analyzed. As can be seen from Figure 3A, there was no significant change in the amount of endocytosed ricin in response to ONO, demonstrating that PLA_2_ activity is not required for ricin internalization.

### 3.3. Ricin Transport to the Endoplasmic Reticulum is Dependent on PLA_2_ Activity

As described in the introduction, ricin is endocytosed and retrogradely transported from early endosomes, through the Golgi apparatus and to the endoplasmic reticulum (ER), before it is translocated to the cytosol where it exerts its cytotoxic effect. The decreased toxicity of ricin in response to PLA_2_ inhibition, in combination with the increased sulfation signal, led us to investigate if the transport of the toxin from the Golgi to the ER might be inhibited. To this end we used a modified ricin molecule, ricin-sulf-2, containing a modified A-chain with *N*-glycosylation sites in combination with radioactively labeled mannose. As mannosylation is an ER-specific modification, this gives a measure of how much of the internalized ricin is being transported to the ER. The data presented in Figure 3B shows a dramatic decrease in mannosylation of ricin-sulf-2 after treatment with 5 µM ONO, indicating that the transport of ricin to the ER is strongly impaired. Quantification shows that there is a close to 90% reduction in ricin mannosylation in the presence of 5 µM ONO. Control experiments show a moderate reduction (~30%) in total mannosylation in response to ONO.

### 3.4. Localization of Ricin and Golgi Markers in the Presence of PLA_2_ Inhibitor ONO

**Figure 4:**
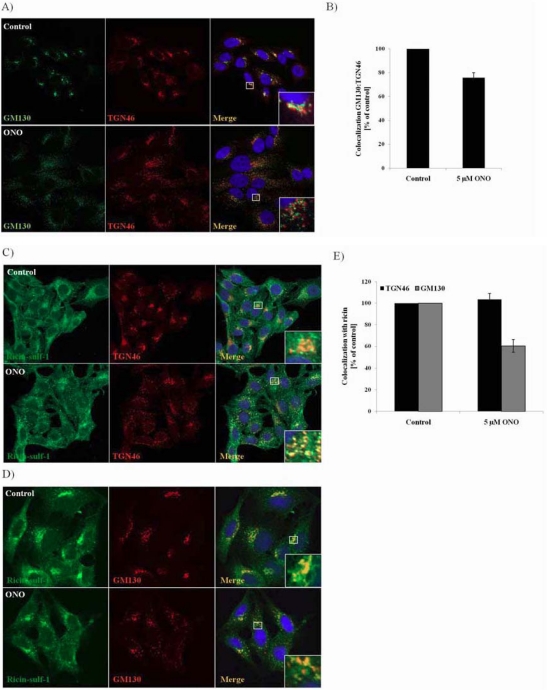
Localization of ricin in response to PLA_2_ inhibition. (**A–B**) After pre-treatment with 5 µM ONO for 30 min, HEp-2 cells were fixed and subjected to immunofluorescence analysis with the indicated antibodies. Colocalization between the Golgi markers GM130 and TGN46 was quantified, and presented as % colocalization compared to control (set to 100%); (**C–E**) After pre-treatment with 5 µM ONO for 30 min, ricin-sulf-1 was added to the cells for 15 min followed by chase for 30 min. The cells were washed with 0.1 M lactose to remove surface-bound ricin, fixed and subjected to immunofluorescence analysis with the indicated antibodies. The relative colocalization was determined, and presented as percentage colocalization relative to control. Representative images are shown, and quantification is from at least three independent experiments with error bars representing standard error of the mean (SEM).

The sulfation and mannosylation data suggest that PLA_2_ activity is essential for Golgi-to-ER transport of ricin, and that PLA_2_ inhibition thereby protects the cells against ricin intoxication. ONO and BEL are known to cause disruption of the Golgi cisternae [23,24], but as ricin is strongly sulfated in the presence of the inhibitors it seems evident that ricin is transported to the Golgi also under these conditions. To get a clearer picture of what is happening to the Golgi in response to PLA_2_ inhibition, and where ricin is localized under these conditions, we performed confocal microscopy studies were we stained cells for ricin in combination with Golgi markers. In Figure 4A we show the localization of the *trans*-Golgi marker TGN46 and the *cis*-Golgi marker GM130 in untreated cells and cells treated with 5 µM ONO. As can be clearly seen from the images, both TGN46 and GM130 staining show that the inhibitor induces fragmentation of the Golgi as reported previously [23,24]. The quantification (Figure 4B) shows that the apparent colocalization between TGN46 and GM130 is decreased in response to ONO, indicating that the inhibitor induces a separation between the TGN and the *cis*-Golgi. 

To analyze the localization of ricin under the same conditions, we stained cells for ricin in addition to TGN46 and GM130 (Figure 4C–E). As can be seen from the images (Figure 4C,D), and more clearly from the quantification of colocalization (Figure 4E), ricin is colocalized with TGN46 to the same extent both in the absence and presence of ONO, while there is a decrease in colocalization with GM130 upon treatment with the inhibitor (from approximately 30% before treatment to 20% after treatment). This indicates that ricin is able to be transported to the TGN, but cannot to the same extent be transported to the *cis*-Golgi. The localization of Stx was not altered in response to ONO (Supplementary Figure S2), demonstrating that the change in localization of ricin is not just a result of the ONO-induced change in Golgi structure. An accumulation of ricin in TGN over time could also be observed by confocal analysis (Supplementary Figure S3), supporting the idea that ricin is inhibited from being transported further along the retrograde transport route. 

**Figure 5:**
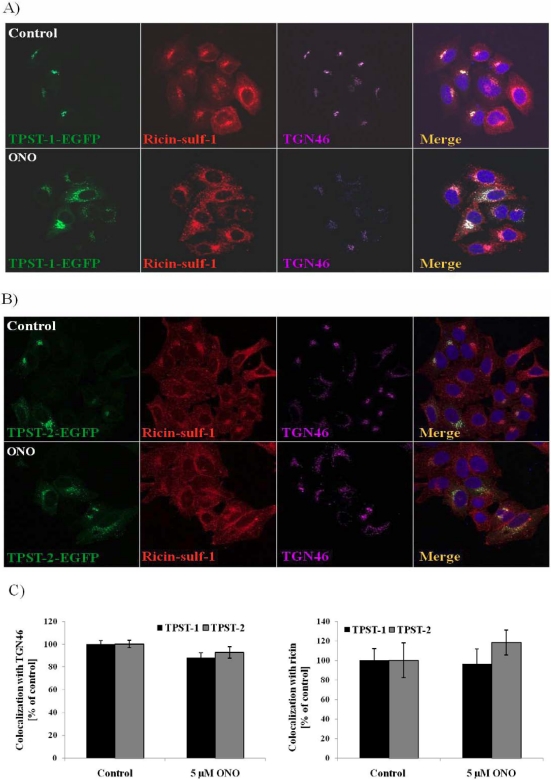
No change in localization of TPST-1 and TPST-2 in response to PLA_2_ inhibition. HEp-2 cells were transfected with pTPST-1-EGFP (**A**) and pTPST-2-EGFP (**B**) plasmids one day before treatment with 5 µM ONO for 30 min. Ricin-sulf-1 was added for 15 min followed by chase for 30 min. The cells were washed with 0.1 M lactose to remove surface-bound ricin, fixed and subjected to immunofluorescence analysis with the indicated antibodies. The relative colocalization was determined by LSM Image Browser software, and presented as percentage colocalization relative to control (**C**). Representative images are shown, and the bar graph shows the average with error bars representing standard error of the mean (SEM).

The increased sulfation seen in response to PLA_2_ inhibition may be due to either more ricin being transported to the TGN, more efficient sulfation in the TGN, or an accumulation of ricin in the TGN as it is inhibited from being transported further on along the retrograde pathway and finally degraded. As the amount of total protein sulfation is not affected by the inhibitors, a general increase in the activity of sulfotransferases does not seem to be a likely explanation. To explore if the treatment with PLA_2_ inhibitor is affecting the localization of the two main sulfotransferases TPST (Tyrosyl Protein SulfoTransferase)-1 and TPST-2, we expressed GFP-tagged versions of these enzymes in HEp-2 cells. The cells were transfected prior to treatment with ONO and exposure to ricin, the localization of the TPSTs was then examined by confocal microscopy, and the colocalization with Golgi-markers and ricin was quantified. As the images in Figure 5A (TPST-1) and 5B (TPST-2) show, the sulfotransferases are mainly localized to the TGN in untreated cells, as expected. Upon addition of ONO, their localization became more dispersed, as observed also for TGN46. The quantification (Figure 5C) shows that the colocalization of TPST-1 and TPST-2 with TGN46 is practically unchanged upon treatment. In addition, colocalization between the sulfotransferases and ricin was also unaffected. These data show that although the Golgi is dispersed upon treatment with ONO, both ricin and sulfotransferases are still localized to the TGN. These data then suggest that the observed increase in ricin sulfation is probably due to either increased transport to the TGN after endocytosis, or accumulation of the toxin in the TGN, or a combination of these. 

## 4. Discussion

In this study we have shown that PLA_2_ activity is important for the retrograde transport of ricin from TGN to the ER, and that a PLA_2_ inhibitor is able to protect cells against the toxic effects of ricin.

The superfamily of PLA_2_ enzymes consists of 15 groups comprising secretory and cytosolic enzymes. In our studies, we have used inhibitors against cytosolic PLA_2_s, and this group can be further divided into Ca^2+^-sensitive (group IV PLA_2_s) and Ca^2+^-insensitive (Group VI, VII and VIII PLA_2_s or iPLA_2_s) isoforms [36]. Both inhibitors used in this work have been widely used in studies of PLA_2_ activity and its role in membrane trafficking and maintenance of the Golgi architecture [21,23,24,25,26,28]. ONO is a broad inhibitor against cytosolic PLA_2_s, and its effect is reversible [37], while BEL on the other hand is reported to be an irreversible inhibitor of iPLA_2_ enzymes [38,39]. In the toxicity assay (Figure 1), we could only use BEL at a concentration of up to 2.5 µM, as higher concentrations of BEL (5 and 10 µM) turned out to inhibit protein synthesis also in the absence of ricin. At a concentration of 2.5 µM, we could not observe any protective effects for BEL, although concentrations even lower than this (1 µM) has been shown to inhibit iPLA_2_ activity [26,29,30,31]. In the sulfation assay, BEL could be used at a concentration of 10 µM without having any effect on total protein sulfation levels, and at this concentration BEL resulted in a significant increase in ricin sulfation. It has been reported that at such high concentrations (10 µM) BEL also inhibits Ca^2+^-dependent PLA_2_ in addition to iPLA_2_[25]. Given the lack of effects on ricin toxicity and sulfation at low concentrations of BEL, we suggest that it is most likely the Ca^2+^-dependent PLA_2_ enzymes that are involved in ricin retrograde transport. More thorough analyses are required to distinguish between the different PLA_2_ groups, for instance by depleting the cells of the separate isoforms by siRNA.

One candidate might be the Group IVA cPLA_2_, cPLA_2_α. This enzyme has been reported to associate preferentially with the Golgi complex, and it was recently demonstrated that cPLA_2_α controls formation of intercisternal tubular continuities, suggesting it to be involved in transport through the Golgi complex [40]. For instance, it was shown that depletion of cPLA_2_α suppressed the intra-Golgi transport of cargo proteins such as vesicular stomatitis virus G (VSV-G) [40]. These authors also explored the possible compensatory role of other PLA_2_ enzymes in cells lacking cPLA_2_α (immortalized lung fibroblasts from cPLA_2_α knock-out mice), and they found that the Ca^2+^-independent Group VIIIA PLA_2_ seemed to play a role. Depletion of Group VIIIA PLA_2_ in these cells resulted in an accumulation of the anterograde cargo VSV-G in the *cis*-Golgi as shown by colocalization with GM130, and a marked delay in protein progression across the Golgi stack [40]. Further studies are needed to examine if these PLA_2_ isoforms are important for retrograde transport of ricin. 

The inhibitor ONO was also used in ricin toxicity assays in other cell lines, and in some cell lines a protective effect could be seen, whereas in others the inhibitor alone was toxic to the cells at the tested concentrations (data not shown). The reason for this difference between cell lines is unknown, but may be due to differences in the repertoire of PLA_2_ enzymes. 

As mentioned in the introduction, PLA_2_ activity is also important for endosomal membrane tubulation [26,27] which could suggest that PLA_2_ inhibition would impair endosome to Golgi transport of ricin. Surprisingly, both the sulfation assay and confocal data show that ricin transport to the TGN is not impaired in presence of the inhibitors. However, ricin transport from TGN to the ER is strongly impaired by ONO treatment, showing that PLA_2_ activity is important for this specific step in the retrograde route. The involvement of PLA_2_ in Golgi-to-ER transport has been demonstrated previously for KDELR-G and TGN38-G, which are chimeric proteins of the thermosensitive luminal domain of VSV-G and the cytoplasmic and transmembrane domains of the KDELR and TGN38, respectively [29]. However, these are chimeric proteins that are transported only at the permissive temperature, and it cannot be concluded that all normal Golgi-to-ER trafficking is PLA_2_ dependent. Also, PLA_2_ does not seem to play any role in the retrograde transport of the bacterial toxin Shiga toxin (Stx) as the inhibitors did not cause any protective effects against this toxin (Supplementary Figure S1A). This clearly shows that proteins following different retrograde transport routes do not necessarily have the same requirements. One interesting study with the PLA_1_ enzyme iPLA_1_γ shows that this phospholipase is involved in transfer of Golgi membrane to the ER in presence of BFA and also transport of the cholera toxin B-subunit from Golgi to the ER [41]. However it is not involved in COPI- and Rab6-dependent retrograde transport represented by ERGIC-53 recycling and Golgi-to-ER transport of Stx [41], demonstrating different requirements for transport of different cargo. One should therefore be careful not to draw general conclusion from studies of single cargos, but study the cargo of interest independently.

## 5. Conclusions

By using inhibitors against cytosolic PLA_2_s we have studied the potential role of PLA_2_ enzymes in the retrograde transport of ricin. The data show that PLA_2_ inhibition protects against ricin challenge. Treatment with inhibitors does not interfere with ricin endocytosis nor does it decrease endosome-to-Golgi transport, however Golgi-to-ER transport is strongly impaired resulting in a protective effect against the toxin. These results emphasize the importance of lipids and lipid composition of membranes for intracellular transport, and gives new insights into the retrograde transport of ricin.

## Supplementary Files

Supplementary File 1: PDF-Document (PDF, 824 KB)
