# Supplementary material for: Role of Phospholipase A2 in Retrograde Transport of Ricin
Source: Toxins (Basel). 2011 Sep 23;3(9):1203–19. doi: 10.3390/toxins3091203 (PMC3202871; doi:10.3390/toxins3091203)

Supplementary Information

Tove Irene Klokk [1], Anne Berit Dyve Lingelem [1], Anne-Grethe Myrann [1] and Kirsten Sandvig [1,2,*]

[1] Centre for Cancer Biomedicine, Department of Biochemistry, Institute for Cancer Research, The Norwegian Radium Hospital, Oslo University Hospital, Montebello, Oslo 0310, Norway; E-Mails: tove.irene.klokk@rr-research.no (T.I.K.); anne.berit.dyve@rr-research.no (A.B.D.L.); anne.grethe.myrann@rr-research.no (A.-G.M.)

[2] Department of Molecular Biosciences, University of Oslo, Oslo 0316, Norway

* Author to whom correspondence should be addressed; E-Mail: ksandvig@radium.uio.no; Tel.: +47-2278-1828; Fax: +47-2278-1845.

Received: 1 July 2011; in revised form: 25 August 2011 / Accepted: 16 September 2011 / Published: 23 September 2011

Abstract: Ricin is a protein toxin classified as a bioterror agent, for which there are no known treatment options available after intoxication. It is composed of an enzymatically active A-chain connected by a disulfide bond to a cell binding B-chain. After internalization by endocytosis, ricin is transported retrogradely to the Golgi and ER, from where the ricin A-chain is translocated to the cytosol where it inhibits protein synthesis and thus induces cell death. We have identified cytoplasmic phospholipase A_2 (PLA_2) as an important factor in ricin retrograde transport. Inhibition of PLA_2 protects against ricin challenge, however the toxin can still be endocytosed and transported to the Golgi. Interestingly, ricin transport from the Golgi to the ER is strongly impaired in response to PLA_2 inhibition. Confocal microscopy analysis shows that ricin is still colocalized with the *trans*-Golgi marker TGN46 in the presence of PLA_2 inhibitor, but less is colocalized with the *cis*-Golgi marker GM130. We propose that PLA_2 inhibition results in impaired ricin transport through the Golgi stack, thus preventing it from reaching the ER. Consequently, ricin cannot be translocated to the cytosol to exert its toxic action.

Keywords: ricin; retrograde transport; phospholipase A_2; Golgi; toxin

---

Cytotoxicity Assay

The toxicity assay for Shiga toxin (Stx) was performed as described in Materials and Methods for ricin, except that after treatment with 2.5 or 5 µM ONO for 30 minutes, increasing concentrations of Shiga toxin [0.1–100 ng/ml] were added.

ShigaB-sulf2

In the sulfation assay and confocal analysis, a modified Stx was used. ShigaB-sulf2 consists of the Shiga toxin B-subunit with two tyrosine sulfation sites added C-terminally. The assays were otherwise performed as described for ricin in Materials and Methods, using anti-Stx antibodies.

Confocal Microscopy of Ricin Time Course

Inhibitor ONO was added 30 minutes before incubation with ricin for 15, 30 or 60 minutes, and the immunofluorescence analysis was performed as described in Materials and Methods for ricin localization studies. Golgi-localized ricin was quantified by identifying ricin colocalizing with the TGN marker TGN46, and then calculating the integrated intensity of ricin staining in this area. The intensity at each time point was normalized to the intensity at 15 minutes to show the accumulation of ricin over time.

Supplementary Data

Figure S1. No change in toxicity or sulfation of Stx in response to ONO. (A) HEp-2 cells were pre-treated with 2.5 or 5 μM ONO for 30 minutes before incubation with Stx for 3 hours followed by [^{3}H]leucine for 20 minutes. Protein biosynthesis was measured by [^{3}H]leucine incorporation, and toxicity curves were generated. The average of three experiments is shown, with error bars representing standard error of the mean (SEM). (B) HEp-2 cells were incubated with radioactive sulfate for 2.5 hours before incubation with ONO for 30 minutes and then ShigaB-sulf2 (3 μg/ml) for another 2 hours. ShigaB-sulf2 was immunoprecipitated from the lysates using anti-Stx antibody, and the precipitate separated by SDS-PAGE. The amount of sulfated Stx (^{35}S-ShigaB-sulf2) was visualized by autoradiography, and the relative change compared to control was quantified. The total amount of sulfated proteins was determined by TCA-precipitation of all ^{35}S-labeled proteins in the lysates. The autoradiogram from one representative experiment is shown, and the bar graph shows the average of three experiments with error bars representing standard error of the mean (SEM).

A)

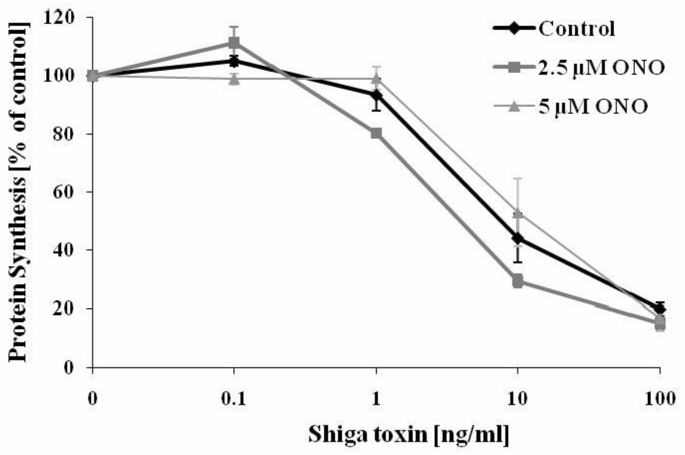

Figure S1. *Cont.*

B)

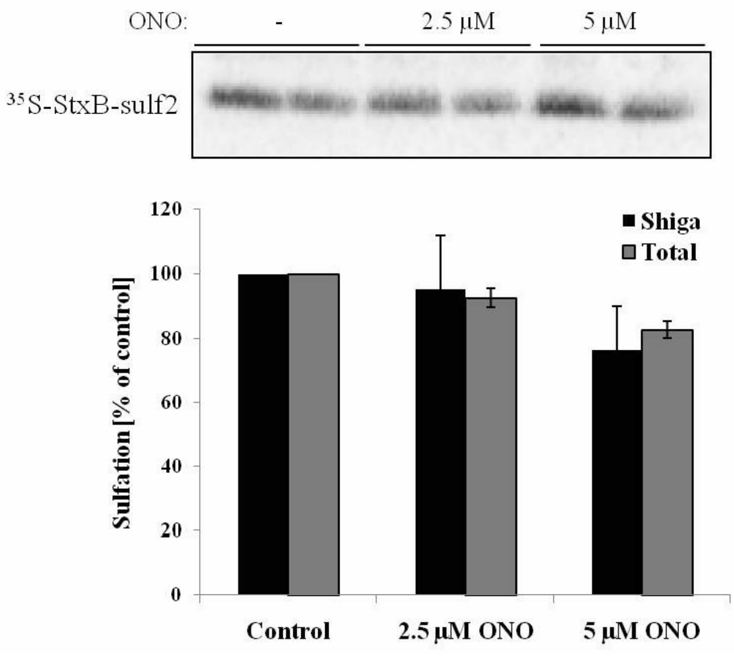

Figure S2. Localization of Shiga toxin in response to PLA_2 inhibition. After pre-treatment with 5 µM ONO for 30 minutes, HEp-2 cells were incubated with ShigaB-sulf2 for 15 minutes followed by chase for 30 minutes. The cells were fixed and subjected to immunofluorescence analysis with the indicated antibodies. The relative colocalization was determined, and presented as percentage colocalization relative to control. Representative images are shown (A-B), and quantification (C) is from at least three independent experiments with error bars representing standard error of the mean (SEM). (D) HEp-2 cells were treated with 5 µM ONO for 30 minutes before the cells were fixed and subjected to immunofluorescence analysis with TGN46 and giantin antibodies. The colocalization between TGN46 and giantin was quantified, and presented as percentage colocalization relative to control. The data is from one representative experiment, and was repeated twice with similar results. At least 30 cells per condition were analyzed, and error bars represent standard error of the mean (SEM).

A)

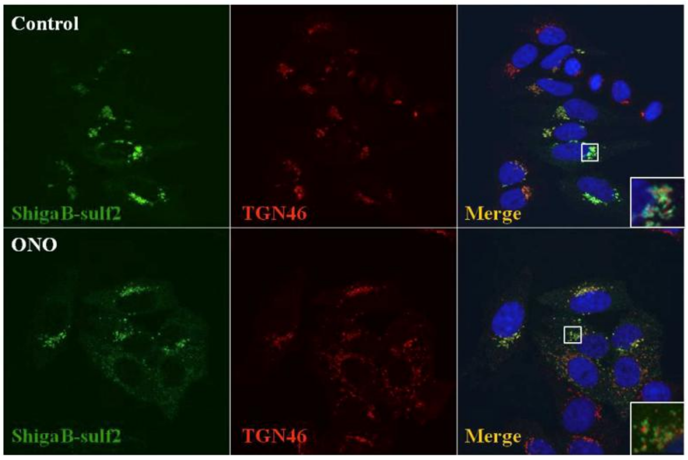

Figure S2. *Cont.*

B)

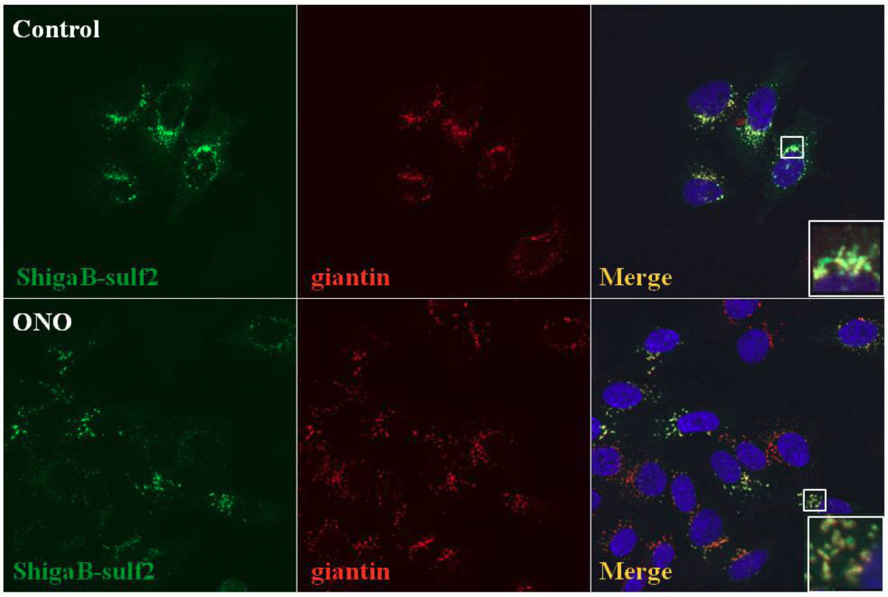

C)

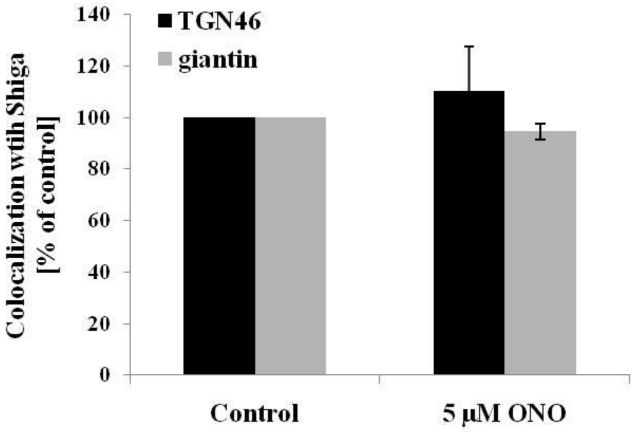

D)

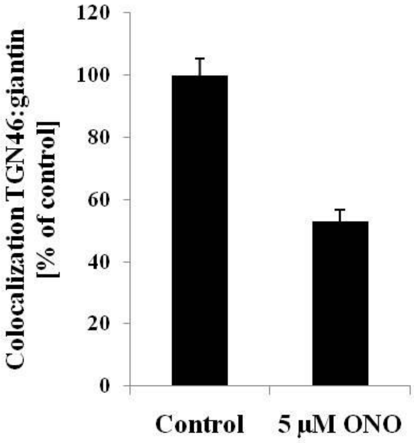

Figure S3. Accumulation of ricin in the Golgi after treatment with ONO. After pre-treatment with 5 µM ONO for 30 minutes, HEp-2 were incubated with ricin for 15, 30, and 60 minutes followed by chase for 30 minutes. The cells were washed with 0.1 M lactose to remove surface-bound ricin, fixed and subjected to immunofluorescence analysis with ricin and TGN46 antibodies. The intensity of ricin colocalizing with TGN46 was quantified, and presented as percentage Golgi-localized ricin relative to levels at 15 minutes. At least 60 cells per condition were analyzed. Images (A) and quantification (B) is from one representative experiment, with error bars representing standard error of the mean (SEM).

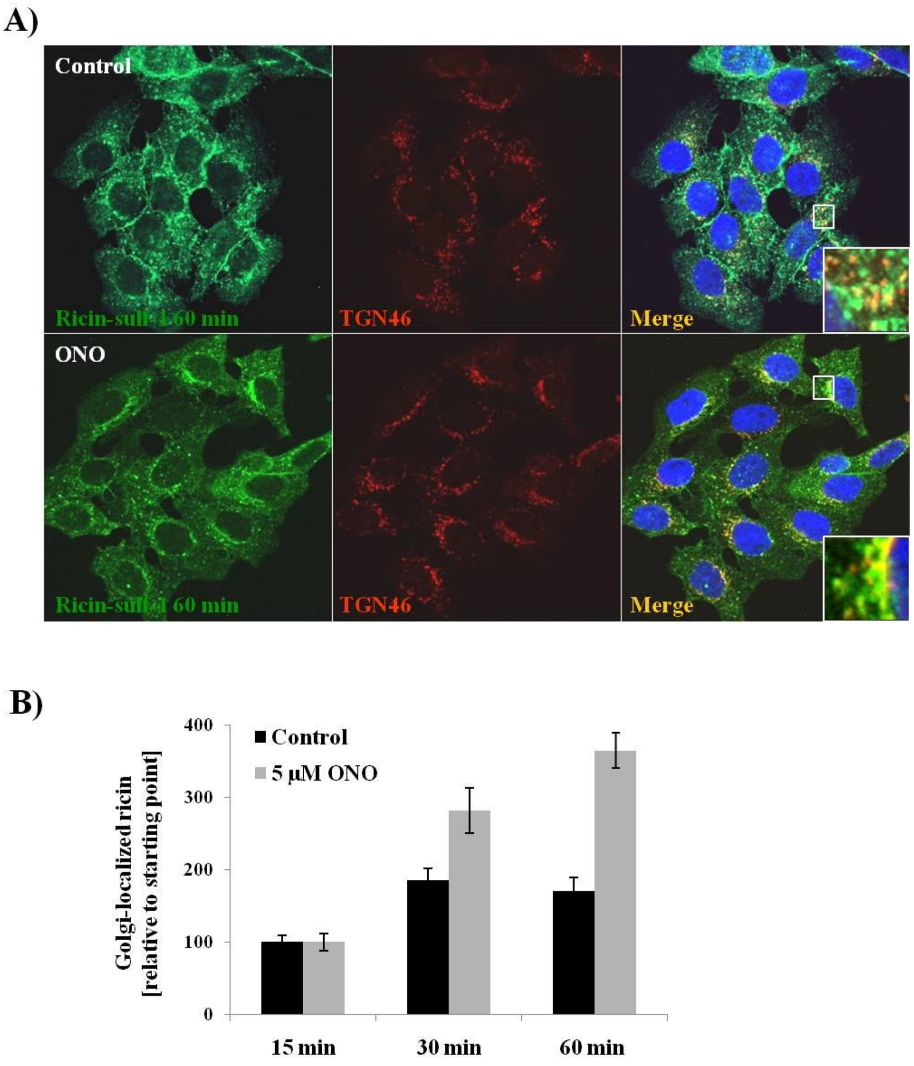

Supplement: Supplementary File 1: — PDF-Document (PDF, 824 KB) [file toxins-03-01203-s001.pdf]
